# Evidence for a specific distortion in perceptual body image in eating disorders: A replication and extension

**DOI:** 10.1371/journal.pone.0313619

**Published:** 2024-11-22

**Authors:** Lise Gulli Brokjøb, Piers L. Cornelissen, Jiří Gumančík, Kristofor McCarty, Martin J. Tovée, Katri K. Cornelissen

**Affiliations:** 1 Department of Psychology, The Artic University of Norway, Tromsø, Norway; 2 Department of Psychology, Northumbria University, Newcastle upon Tyne, United Kingdom; Union College, UNITED STATES OF AMERICA

## Abstract

A core feature of eating disorders, such as anorexia nervosa, is an overestimation of body size. A key question is whether this overestimation arises solely from body image concerns typical in eating disorders, or if there is an additional perceptual disturbance. To address this question, we applied a two-component model of body size estimation that has been thoroughly replicated in the body image literature concerning healthy individuals. This model shows statistically independent, additive effects on body size estimates of: a) body image concerns, and b) a perceptual component known as contraction bias. Here body image concerns were defined by a principal components analysis (PCA) of psychometric tasks including the: Eating Disorder Examination Questionnaire, Beck Depression Inventory, Body Shape Questionnaire, Rosenberg Self-Esteem Scale, and Sociocultural Attitudes Towards Appearance Questionnaire-4. The PCA identified three components referred to as PSYCH, FAMPEER, and ATHIN. We investigated the influence of age, personal body mass index (BMI), and these three components on body size estimation in 33 women with a current or past history of eating disorders and 100 healthy controls. Low-BMI control participants overestimated their size, while high-BMI controls underestimated their size, exhibiting the expected normal perceptual contraction bias. However, the women with a history of eating disorders showed no evidence of contraction bias, suggesting a different processing of perceptual aspects of body size estimation compared to controls. We discuss two putative mechanisms that can explain these differences in accuracy of personal body size estimation.

## Introduction

### Eating disorders and body image

Eating disorders, such as anorexia nervosa (AN) and bulimia nervosa (BN), pose a significant public health concern due to their complex causes, treatment challenges, and profound impact on individuals’ lives [1–5]. A key feature in eating disorders is a disturbance in body image, a multifaceted construct that encompasses how individuals perceive, evaluate, and emotionally respond to their own bodies [6–8]. Body image disturbances are closely linked to anxiety and depression and have a detrimental effect on self-worth and mental well-being [7–10]. Persistent body image disturbance post-recovery is a key predictor of eating disorder relapse [6,11,12]. Therefore, to tailor clinical interventions more effectively, we need a more detailed understanding of body image and how it may become disturbed.

### Perceptual and attitudinal body image in healthy individuals

The conceptualization of body image by Cash and Deagle [13] proposes separate and independent attitudinal and perceptual dimensions. This framework is useful for the current purposes and has direct empirical support [14]. Perceptual body image refers to the accuracy with which an individual can discern their body’s physical dimensions [13], while attitudinal body image encompasses the attitudes and cognitive responses one has towards their body, often manifesting in body satisfaction levels. To better understand body image disturbances in eating disorders, it is essential to first examine body image perception in healthy individuals to establish a point of comparison. In healthy adult women, a consistent phenomenon affecting their perceptual body image is contraction bias [15]. This bias is a normal feature of our perceptual systems and affects how we estimate the size of objects. When applied to bodies, it is assumed that we compare the body currently being viewed (one’s own or another’s) to an internal reference distribution formed from previously encountered body sizes. The most accurate size estimations occur when the body being assessed is similar in size to the average of this reference distribution [16–18]. However, as the size difference grows between the body being observed, and the reference average, the accuracy of these estimations decreases. This leads to a progressive compression of the response range, leading to bodies smaller than the reference average judged to be larger and larger bodies to be smaller. This contraction bias in judgements of body size has been consistently observed in healthy individuals [19–25].

The attitudinal component of body image encompasses both emotional reactions and cognitive assessments related to one’s body. This includes how a person feels about their appearance (affect) and what they believe about their looks (cognitions/attitudes [26]). The more negative an individual’s attitudes about their own body, the larger they estimate their own body size to be. This relationship is simple, linear and is statistically independent from the perceptual phenomenon of contraction bias [14,17,18]. Attitudinal body image disturbances are typically characterized as a multidimensional construct encompassing psychological concerns regarding body size and weight and dysfunctional attitudes towards eating. These are commonly measured using a number of psychometric tasks such as: a) the Eating Disorders Examination Questionnaire, EDEQ [27], b) the Body Shape Questionnaire, BSQ [28], c) low self-esteem measured by the Rosenberg Self-Esteem Scale, RSE [29], and d) the tendency towards depressive symptoms measured by the Beck Depression Inventory, BDI [30].

### Quantitative representation of perceptual and attitudinal body image in healthy women

**Fig 1:**
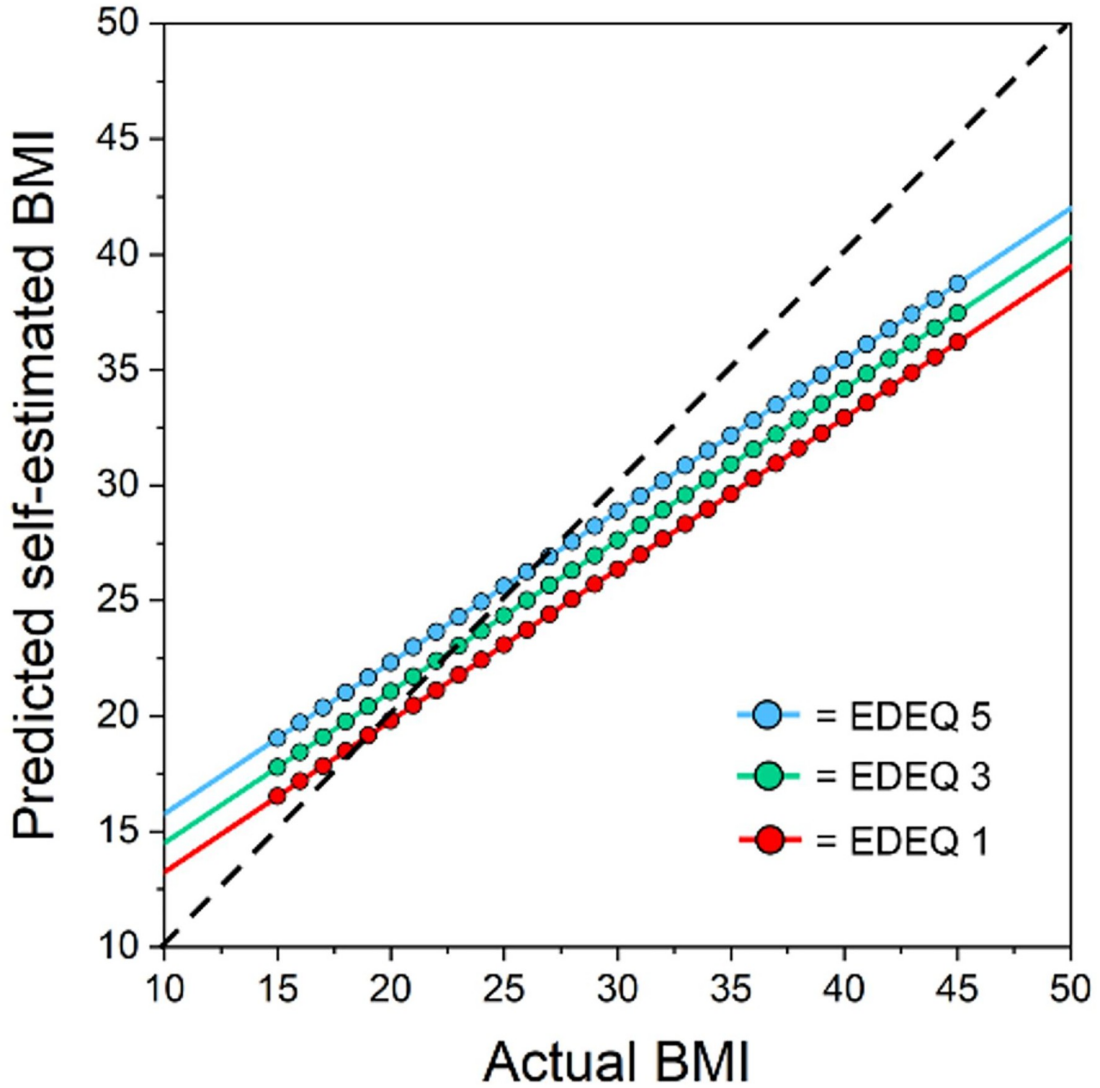
Sketch plot of estimated BMI (y-axis) as a function of actual BMI (x-axis). The perceptual effect of contraction bias is apparent as a consistent regression slope of < 1 across all three regression lines: Actual BMIs below the population average are overestimated, actual BMIs above the population average (i.e.,~25) are underestimated, and actual BMIs close to the population average tend to be accurately estimated. Meanwhile, the attitudinal effect, captured by global EDEQ scores, is reflected in the systematic upward shift of the three regression lines as the EDEQ scores increase, reflecting increasing attitudinal body image concerns. This suggests that for any given actual BMI, the self-estimated BMI increases by a fixed amount according to the EDEQ score.

Using this conceptualization of body image, the perceptual and attitudinal body image dimensions, and their inter-relationship, can be effectively illustrated by a sketch graph Fig 1. The data for such a graph might come from a typical body size estimation task administered to a large sample of individuals varying in actual body size. Across several trials, each participant is shown images representing a large range of BMIs and asked to choose the image that most accurately represents their own body size/shape. Overall, this graph reveals the pattern of stimulus response pairings for the sample, which we assume generalizes to the population at large. It shows estimated body size in BMI units (the response) on the y-axis, plotted as a function of actual body size (the stimulus) BMI units on the x-axis. The dashed line, with a slope of 1, is the line of equality which represents the theoretical scenario where women’s estimations of their BMI are perfectly accurate (i.e. where response = stimulus). However, the actual real-world data for healthy women shows a slope less than 1, illustrating the effect of contraction bias, which compresses the stimulus-response range. The point where the regression line appears to rotate clockwise in relation to the line of equality is close to the population average BMI for women (~25 BMI units in the UK for women 18–45 [31]). In essence, this graph illustrates that low actual BMIs (e.g., ~ 16) tends to be overestimated, and high actual BMIs (e.g., ~35) are under-estimated. Actual BMIs close to the population average (e.g., ~25) are estimated most accurately. This model also illustrates the effect of body image concerns as indexed by scores on the Eating Disorder Examination Questionnaire (EDEQ [27]). The regression lines between actual BMI and self-estimated BMI are plotted at three different levels of body image concerns: ‘low’ (EDEQ 1), ‘middle’ (EDEQ 3), and ‘high’ (EDEQ 5). The parallel nature of these lines indicates that the influence of body image concerns is statistically independent from the perceptual influence of contraction bias. Therefore, for any given actual BMI, a change in the level of body image concern (e.g. from ‘low’ to ‘middle’ EDEQ) results in a consistent increase in estimated body size so that the vertical distance between the two lines remains constant. Thus, this model effectively demonstrates how both attitudinal and perceptual factors in body image can be quantitatively assessed and visually interpreted, providing clear and measurable insights into their impact.

### Unique perceptual body image disturbances in clinical groups?

A core diagnostic feature of people with eating disorders, such as women with AN, is an overestimation of body size [12,32–34]. A key question is whether this overestimation arises solely from body image concerns typical in eating disorders (i.e. disturbance to the attitudinal component), or if there is an additional perceptual disturbance component unique to people with eating disorders.

To address this question, Cornelissen et al. [17,18] used psychophysical methods to have women with eating disorders and healthy controls estimate their own body size. To reliably test whether the regression slopes of self-estimated BMI on actual BMI were the same or different between the two groups, it was important that the eating disorder sample included participants with a wide range of actual BMIs. This meant that these individuals no longer fell within the typical AN diagnostic criteria, which include a significant low body weight relative to age, sex, developmental trajectory, and physical health (DSM-V). For this reason, the samples were labelled as having anorexia spectrum disorder (ANSD) in Cornelissen et al. [17], which amounted to a current eating disorder diagnosis that did not necessarily meet the low-weight criteria of AN. In an attempt to recruit an eating disorder sample with a wider BMI range and investigate any persistent eating disorder symptoms post-recovery, Cornelissen et al. [18] included women with a current or past history of eating disorders.

As depicted in Fig 1, the impact of attitudinal body image disturbances on self-estimated BMI was equivalent across women with ANSD and healthy controls. However, there was a notable difference in the slopes of the regression lines between the two groups. Healthy controls performed as illustrated in Fig 1. In contrast, the regression slope for women with ANSD estimating their BMI in relation to their actual BMI was significantly greater than 1. This pattern indicates an accelerating overestimation of body size in the group with ANSD. For individuals with ANSD who had a BMI near ~16, self-estimated BMI was relatively accurate. However, for BMIs higher than this in the group with ANSD, overestimation systematically increased as a function of increasing actual BMI. This effect, which was statistically independent from the effect of attitudinal body image disturbances, strongly suggests differences in the ways that women with ANSD process the perceptual aspects of body size and shape.

### The current study

#### BMI range discrepancy

While the study design in Cornelissen et al. [17,18] required comparable ranges of actual BMIs among participant groups, this ideal was not fully achieved. Specifically, in Cornelissen et al. [17], the control group’s BMI ranged from 17 to 44 (*M* = 24.01, *SD* = 5.03), whereas the BMI range for the ANSD group was more restricted, spanning only from 15 to 25 (*M* = 20.89, *SD* = 2.84). This disparity raises the possibility that the distinct perceptual disturbances observed in the ANSD group might not be attributable to their eating disorder status but could instead be due to a statistical artefact resulting from the narrower BMI range in this set of participants. In short, this range constriction might have led to an unreliable estimation of the regression weights. To address this concern, Cornelissen et al. [17] reanalysed the data, capping the upper BMI limit for all participants at 22.3, thereby ensuring that the BMI range across both groups was the same. Even with this adjustment, the significant difference in regression slopes between the two groups remained. Nevertheless, uncertainty remains. For this reason, the current study uses a wider range of actual BMIs and better matches the actual BMI ranges across participant groups. This approach provides a stronger test to determine whether the perceptual disturbances in the eating disorder group are indeed a function of the eating disorder or are produced by differences in the BMI ranges represented.

#### Sociocultural attitudes towards appearance

The principal sociocultural explanation of body dissatisfaction and disordered eating, the tripartite influence model, emphasizes the importance of social pressure to be thin, derived from three sources: family, peers, and media [35,36]. Individuals vary both in their exposure to these pressures and the extent to which they internalize these pressures (i.e., the degree to which the appearance-related messages are judged to be important and relevant to themselves). This internalization, together with social comparison processes, is proposed as the link between societal body ideals and the development of body dissatisfaction [37–41]. Body dissatisfaction arises because most people do not match these appearance ideals but feel pressure to conform to this archetype.

Neither the Cornelissen et al. [17,18] studies controlled for sociocultural attitudes towards appearance. Given that phenomena such as thin-ideal internalization are risk factors for body image disturbances, it is at logically possible that they may also influence the slope of the regression of self-estimated BMI on actual BMI. Therefore, the current study included the Sociocultural Attitudes Towards Appearance Questionnaire (SATAQ) to test for this possibility.

#### Emotional significance of deciding “larger/smaller than” another body

In Cornelissen et al. [17,18], participants estimated their body size using a psychophysical yes/no task. In each trial, participants were shown an image of a female body avatar and asked to determine whether the avatar was larger or smaller than their own body. While both studies accounted for attitudinal factors as outlined above, these measures (EDEQ, BSQ, RSE, and BDI) might not fully encompass participants’ deeply ingrained attitudes towards thinness, fatness, and muscularity, as SATAQ was not included. For instance, the emotional impact of considering oneself to be “larger” or”smaller than” another might differ significantly for individuals with eating disorders compared to individuals without eating disorders, even if both groups experience similar personal challenges relating to food and body image. To address this, the current study employed a method of adjustment (MoA) task that avoided responses carrying such loaded terminology. Instead, participants were simply asked to select the body shape that most closely matched their own, thereby reducing potential bias in their responses due to emotional factors triggered by language usage.

In summary, the current study serves both as a replication and extension of Cornelissen et al. [17,18]. We aimed to replicate the differences in perceptual body image identified in these previous studies by improving the design in the following ways: a) ensuring a more accurate alignment of actual BMI ranges across participant groups, b) incorporating measures to control for sociocultural attitudes towards appearance, and c) implementing a refined psychophysical method for body size estimation that avoided emotionally loaded language in the responses.

## Materials and methods

The study was pre-registered and the data is available to download at https://osf.io/nhsyv/.

### Ethics

Ethical approval for this study was granted by the Department of Psychology Ethical Committee at Northumbria University. The data were collected between 18th June 2021 and 26th July 2022 (Ethical approval ref: 33020).

### Sample size

Statistically, the central finding of Cornelissen et al. [17] that supports a difference in the way healthy controls and women with ANSD perceive body size was a significant interaction between BMI and participant group in predicting self-estimated body size. In this replication study, we followed the same design as Cornelissen et al. [17] by comparing ~100 healthy controls to ~30 women with ANSD, maintaining a sample size ratio of ~ 3:1. We used G*power [42] to estimate the required sample size for a linear bivariate regression model with two groups (i.e., healthy controls and women with ANSD in a sample size ratio of 3:1) for whom we sought significantly different slopes. We used the *SD* of self-estimated BMI (y) and actual BMI (x), as well as the correlation between the two extracted from both participant groups in Cornelissen et al. [17]. At a power of 0.8, and alpha levels set to .05 and .01, G*power estimated the required sample sizes for the current study. For an alpha of 0.05, the estimated sample sizes are 63 healthy controls and 21 women with a history of eating disorders. For an alpha of 0.01, the estimated sample sizes are 102 healthy controls and 34 women with a history of eating disorders.

### Participants

The current study recruited adult women with and without a current or past history of eating disorders. All participants were recruited through opportunity sampling. Eligible individuals were female as assigned at birth, aged 18 years or older, and had sufficient reading skills in English. Those who self-reported a history of eating disorders were asked: a) was the diagnosis made by a clinically qualified professional? b) what was the diagnosis given? c) how long ago was the diagnosis made? d) would you identify as recovering or recovered from an eating disorder? e) have you ever experienced a relapse for an eating disorder? f) when was your last relapse? and g) how many relapses have you experienced (roughly)? Participants were recruited using social media and the Northumbria University’s SONA system. The study recruitment period spanned from June 2021 to July 2022. To maximize recruitment efforts participants could take part in the research either online or in person at the university laboratory. This dual approach was employed as the questionnaires and psychophysical tasks have shown congruent outcomes when used online and in person [21]. From the 241 women who showed initial interest in the study, we were able to recruit 133 women to the study who gave informed consent and who provided complete datasets on all tasks. Of these, 52 healthy controls participated in the laboratory study, and 48 participated online. Among the 33 women with a history of eating disorders, 17 participants took part in the laboratory study, and 16 participated online.

## Materials

### Psychometric and anthropometric measures

A number of psychometric measures were used to establish participants attitudinal body image. The particular questionnaires were chosen to be consistent with previous research.

#### Eating Disorder Examination Questionnaire, EDEQ [27]

The EDEQ is a self-report measure comprising four subscales: 1) the Restraint subscale with 5 items measuring restrictive nature of eating; 2) the Eating Concern subscale measuring preoccupation with food and social eating; 3) the Weight Concern subscale with 5 items measuring dissatisfaction with body weight, and 4) the Shape Concern subscale with 8 items measuring dissatisfaction to body shape. Participants report how many days within the past 4 weeks (28 days) they have experienced the concerns/behaviours outlined in each item. e.g., ‘On how many of the past 28 days have you been deliberately trying to limit the amount of food you eat to influence your shape or eight (whether or not you have succeeded)?’ Responses are given on a 7-point scale ranging from 0 (no days) to 6 (every day). For each subscale, the sum of the scores for the relevant items is divided by the number of items in that subscale to give a score ranging between 0 to 6. The global score is the average of the four sub-scores, with higher scores indicating greater disordered eating pathology.

Additionally, the measure collects frequency information of eating disorder pathology, e.g., ‘Over the past 28 days, how many times have you exercised in a “driven” or “compulsive” way as a means of controlling your weight, shape or amount of fat, or to burn off calories?’ However, these frequency data were not used in the data analysis in the current study. The reliability coefficient in the current sample indicated high reliability with α = 0.96 for laboratory data collection and α = 0.95 for online data collection.

#### Beck Depression Inventory, [30]

The BDI consists of 21 items that assesses the severity of depressive symptoms. The measure includes items describing cognitive symptoms, such as guilt, and physical symptoms such as fatigue. Each item uses a response scale that ranges from 0 to 3. The total score is created by summing all item responses, resulting in a score ranging from 0 to 63. This score can be used to indicate symptom severity, ranging from 0–10 (normal mood), 11–16 (mild mood disturbance), 17–20 (borderline clinical depression), 21–30 (moderate depression), 31–40 (severe depression), and 40 and above (extreme depression). The reliability coefficient in the current research indicated high reliability, with α = 0.89 for laboratory data collection and α = 0.92 for online data collection.

#### Body Shape Questionnaire, BSQ [28]

The BSQ consists of 16 items that measure participants’ negative attitudes towards their body size, shape, and weight. Examples of items are ‘Have you been so worried about your shape that you have been feeling you ought to diet?’. Items are rated on a 6-point response scale, from 1 (never) to 6 (always). All item responses are summed to a total score which indicating the severity of body shape concerns, ranging from <38 (no concern with shape), 38–51 (mild concern with shape), 52–66 (moderate concern with shape), and >66 (marked concern with shape).). The reliability coefficient in the current sample indicated high reliability, with α = 0.95 for both laboratory and online sample.

#### The Rosenberg Self-Esteem Scale, RSE [29]

The RSE consists of 10 items measuring self-esteem. Each item is rated on a 4-point scale ranging from ‘strongly agree’ to ‘strongly disagree’. Five items are negatively worded (e.g., ‘All in all, I am inclined to feel that I am a failure’), and five are worded positively (e.g., ‘I feel that I have a number of good qualities’). The negatively worded items require reverse scoring before the scores are summed. The total scores range from 0–30, with higher scores indicating more severe difficulties with self-esteem. The reliability coefficient in the current sample indicated high reliability, with α = 0.86 for laboratory data collection and α = 0.87 for online data collection.

#### Sociocultural attitudes towards appearance Questionnaire-4, SATAQ-4 [40]

The SATAQ-4 is a 22-item questionnaire that measures internalisation of appearance ideals and appearance related pressures. It assesses attitudes towards one’s appearance using five subscales: two for internalisation of ideals (thin/low body fat and athletic/muscular dimensions) and three for pressures (family, peers, and media dimensions). Each item is rated on a five-point Likert scale, with response options ranging from 1 (definitely disagree) to 5 (definitely agree). Higher scores on each subscale indicate greater internalisation and acceptance of societal appearance ideals, as well as greater pressures from family, peers, and media. The two internalisation sub-scale scores each range from 5 to 25, and the three pressures subscale scores each range from 4 to 16. The reliability coefficient for the current sample indicated high reliability, with α = 0.88 for both online and in-lab.

#### Body Mass Index (BMI)

In the laboratory, participants body weight was measured using a set of calibrated scales and their height was measured using a stadiometer. For the online recruitment, participants were provided with written and visual instructions on how to measure their weight and height effectively. This included instructions such as taking off their shoes and heavy clothing such as jackets or jumpers when measuring weight, standing as close to the wall as possible, and to be mindful of not slouching when measuring height. Participants conducted their own measurements and inputted this into the survey, which was used to calculate BMI.

#### Demographics and eating disorder status

To collect demographics, participants were asked their age, gender, ethnicity, country of birth, country of current residence, and employment status.

To assess eating disorder history, participants were asked about any past or current eating disorder diagnoses. Specifically, whether they had ever been diagnosed with an eating disorder by a health professional, and if so, what specific diagnosis they had received, and how long it had been since they received the diagnosis. Participants were also asked whether they identified as recovering or having recovered. Lastly, they were asked if they had ever experienced a relapse of an eating disorder, and if so, how long since their last relapse and how many relapses they had experienced.

### Stimuli and psychophysical measurement

#### Stimuli

Participants estimated their own body size/shape using a MoA task. The avatar stimuli were generated using a 3D Computer-Generated Imagery (CGI) model of an adult female (for examples, see [43]). This model’s dimensions and BMI calibration were based on average data for UK women obtained from the Health Survey for England [44,45]. Using DAZ v4.8 software (2017), we generated a sequence of images with BMI values ranging from 12.5 to 44.5 units. Images were rendered using LuxRender software, Version 2.4 [46]. This approach produced photo-realistic images that preserve the female model’s appearance across a wide range of BMI values and demonstrate realistic BMI-dependent body shape changes. To calculate BMI values for each of the avatar bodies, we used the Health Survey for England datasets to create calibration curves between waist and hip circumferences and height derived from over 5000 women aged between 18 and 45. Because our CGI models exist in an appropriately scaled 3D environment, with the height of our models set to the average height of women in England (1.62 m), we can measure their waist and hip circumferences, and compare these with our HSE calibration curves to calculate their BMI (for further details see [43,47]).

#### Psychophysical measurement

Psychophysical measurements were taken using a MoA task, constructed using the PsychoJS JavaScript library, part of PsychoPy3 [48]. This task, hosted online at pavlovia.org, needed to be completed using a desktop or laptop PC (i.e., not a tablet or mobile phone) and was always presented full screen. To ensure adherence to this for online participants, the software was programmed to identify the platform used, and redirected participants accessing the task from a phone or tablet to another page requesting participants to access the task from a desktop or laptop PC. Each trial of the MoA task began with the participant clicking a central white plus sign on a black screen to turn it green. This action triggered the appearance of a female avatar scaled to 70% of the device’s screen height while maintaining the original image aspect ratio. The initial appearance of the avatar was randomized between its lowest and highest BMI settings from one trial to the next. Below the stimulus image appeared a horizontal red rectangle which stretched to the full width of the screen (see https://osf.io/nhsyv/). Participants were asked to move the mouse cursor into the red zone, which immediately turned green, and caused the mouse cursor to become invisible. Once in the green zone, they were encouraged to move the mouse leftward to reduce the body size of the image and rightward to increase its body size. In this way, participants used horizontal mouse movements to find a body size/shape that they believed best represented their own body size and clicked the left mouse button to register their choice. This action triggered the reappearance of the white plus sign in the middle of a black screen and initiated the next trial. Pilot testing showed that this arrangement removed any spatial cues that participants might otherwise rely on to remember where to move the mouse to from one trial to the next and forced them to focus only on body size change caused by their horizontal mouse movements.

### Procedure for online study

Participants who carried out the study online were provided with a link to Qualtrics. They were first presented with a study information sheet and the opportunity to provide informed consent for study participation. If consenting, by clicking on the appropriate field, participants were instructed to create a unique, memorable code word to identify them should they later wish to withdraw from the study. Next, participants were presented the questions seeking demographic information, the request to their input height and weight as well as information about their eating disorder status. They then carried out 20 trials of the MoA task to assess their perceived body size. Following this, participants filled out the psychometric questionnaires, and then completed another 20 trials of the MoA task. Finally, participants were presented with a debrief sheet.

### Procedure for laboratory study

Participants who took part in the laboratory study were first welcomed and acclimatized to the laboratory environment. They were then presented the study information sheet and offered the opportunity to provide written informed consent to take part in the study, which was witnessed by research assistants. If consenting, participants had their height and weight measured. They were then prompted to provide demographic information and information about eating disorder status. Following this, participants completed the psychometric questionnaires, and then carried out 40 trials of the MoA task. Finally, they were presented with a debrief sheet. As this study took place during the COVID-19 pandemic, necessary sanitation and personal protective equipment were provided all throughout participation, with the laboratory equipment being sanitised after each participant.

### Data analysis

Data analysis was conducted using SAS v9.4. Initial data examination included univariate statistics to describe the participant characteristics. Pearson correlations were calculated to assess the relationships among psychometric variables. Given significant correlations, a principal components analysis (PCA) with varimax rotation was performed using PROC FACTOR to identify significant latent variables in the psychometric data. The Kaiser–Meyer–Olkin (KMO) measure confirmed sampling adequacy, and factors with eigenvalues greater than 1 were retained.

Subsequently, a linear mixed effects model was employed using PROC MIXED to predict self-estimated BMI. The model included fixed effects for group (healthy controls vs. eating disorder history), age, actual BMI, and the latent variables derived from PCA (PSYCH, FAMPEER, ATHIN), as well a data collection method (in-lab vs. online). Interaction terms were tested to explore differential effects across groups. Fixed effects were retained in the model if they gave rise to a) significant Type III tests of fixed effects, and b) produce a significant reduction in -2 Log Likelihood. If an isolated fixed effect was non-significant, but nevertheless contributed to a significant interaction term, it was also retained. Overall model fit was assessed by the proportion of variance explained (*R*^*2*^). This structured approach allowed us to systematically analyse the data, accounting for both individual differences and psychometric variables, ensuring robust and reliable statistical inferences.

## Results

### Univariate statistics

We recruited 100 healthy women as controls, and 33 women with a history of eating disorders. Of this sample of 133 women, 93.2% were White, 3.0% Asian, 1.5% Black, 0.75% Mixed race, and 1.5% preferred not to say. For country of origin, 57.6% were from the UK, 23.5% from Europe, and 18.9% outside of Europe. For country of residence, 84.9% were from the UK, 1.5% from Europe, and 13.6% outside of Europe. Regarding employment, 55.3% were students, 35.6% in full or part-time employment, 3.8% described themselves as unemployed, and 5.3% preferred not to say.

With regard to gender, 99.0% of women with eating disorders self-reported as cisgender, while 1.0% did not. Similarly, 96.9% of healthy controls self-reported as cisgender, while 3.1% did not. In terms of sexual orientation, we sought self-reports for the following categories: asexual, bisexual, heterosexual, homosexual/gay/lesbian, other, pansexual, and queer. The respective percentages for these categories self-reported by women with eating disorders and healthy controls were, respectively: 6.3%, 40.6%, 40.6%, 3.1%, 0.0%, 6.3%, 3.1%, and 3.1%, 15.3%, 72.4%, 5.1%, 1.0%, 2.0%, 1.0%.

Among the 33 women with eating disorders, 19 had a history of anorexia nervosa, six a history of bulimia nervosa, and eight a history of other specified feeding or eating disorder (OSFED) consistent with the atypical anorexia definition in the Diagnostic and Statistical Manual of Mental Disorders, Fifth Edition (DSM-5). 87.5% of the women with a history of eating disorders self-reported that they were either recovered or recovering, and 89.7% of them self-reported one or more instances of relapse. For these 33 individuals, Table 1 describes the history of their eating disorders over time.

**Table 1:** Eating disorder histories over time.

	*Mean*	*Median*	*SD*
Number of years since first diagnosis	5.32	4.00	3.94
Number of months since last relapse	12.39	12.00	10.19
Number of relapses	1.93	2.00	1.54

Participant characteristics are shown in Table 2. The last column of this showing pairwise comparisons between healthy controls and those with a history of eating disorders.

**Table 2:** Participant characteristics.

	Healthy controls N = 100			ED history N = 33			P-value
	*M*	*SD*	Range	*M*	*SD*	Range	
Age (years)	22.93	7.19	18–64	22.48	4.36	18–35	
BMI	23.71	4.81	16.53–40.77	23.5	5.22	17.44–40.72	
BSQ	51.56	20.07	17–89	62.18	20.95	23–96	.01
RSE	24.48	4.46	11–40	24.48	3.10	17–30	
BDI	24.74	14.40	0–58	31.18	13.89	13–70	.03
EDEQ restraint	2.22	1.67	0–6	2.90	1.75	0.4–6.8	.05
EDEQ eating concern	1.78	1.35	0–5	2.91	1.60	0.6–6.6	< .001
EDEQ weight concern	2.87	1.73	0–6.60	3.72	1.77	0.6–6.4	.02
EDEQ shape concern	3.45	1.83	0–6.75	4.09	1.93	1–6.75	
EDEQ global	2.58	1.48	0–5.59	3.41	1.61	0.94–6.61	< .01
SATAQ int thinness/low body fat	16.88	4.99	5–25	19.12	4.96	3–25	.03
SATAQ int muscular/athletic	12.90	4.80	5–25	14.61	5.07	5–25	
SATAQ family pressures	9.77	5.05	4–20	10.12	4.79	4–20	
SATAQ peer pressures	7.11	4.27	4–20	6.76	3.17	4–17	
SATAQ media pressures	15.42	4.72	4–20	17.42	3.00	8–20	.006

### Multivariate statistics: Controls versus women with an eating disorder history

In the multivariate analysis, we aimed to determine whether the results of previous studies could be replicated using the current stimulus set, both online and in the laboratory. Specifically for controls, we examined whether a regression of self-estimated BMI on actual BMI showed: (a) a perceptual contraction bias, and (b) an independent contribution to self-estimated BMI from participants’ psychometric performance. For women with a history of eating disorders, we tested whether a regression of self-estimated BMI on actual BMI showed accelerating overestimation as participants’ actual BMI increased. To prevent variance inflation in the statistical models, we initially tested for collinearity among the psychometric variables.

Given the substantial and significant Pearson correlations between EDEQ sub-scores, BDI, BSQ, RSE, and the SATAQ sub-scores, we carried out a principal components analysis (PCA) with varimax rotation using PROC FACTOR in SASv9.3 (SAS Institute, North Carolina, US) to identify significant latent variable(s) in the psychometric data. The factor scores from these latent variable(s) were used in the statistical models. The Kaiser–Meyer–Olkin (KMO) measure of sampling adequacy, which indicates the degree of diffusion in the pattern of correlations, was 0.90, suggesting an acceptable sample. Three factor had eigenvalues greater than Kaiser’s criterion of 1 (i.e., 5.74, 1.36, and 1.14), explaining 69% of the variance. The scree plot showed an inflexion, i.e., Cattel’s criterion, which also justified retaining just the three factors. The residuals were all small, with overall root mean square off-diagonal residual of 0.07, indicating that the factor structure explained most of the correlations. The factor loadings for the three principal components (PC) are shown in the last three columns of Table 3.

**Table 3:** Pearson correlations between psychometric subscales, together with PCA factor loadings. Loadings <0.4 are not shown.

	EQsc	EQwc	EQeat	EQres	BSQ	BDI	RSE	SQfam	SQpeer	SQmedia	SQathl	PC1	PC2	PC3
EDEQsc	-											0.88		
EDEQwc	0.93***											0.87		
EDEQeat	0.81***	0.81***										0.87		
EDEQres	0.65***	0.68***	0.68***									0.83		
BSQ	0.88***	0.88***	0.76***	0.61***								0.81		
BDI	0.53***	0.48***	0.59***	0.42***	0.43***							0.60		
RSE	0.44***	0.40***	0.44***	0.28**	0.44***	0.39***							0.46	
SATAQfam	0.40***	0.38***	0.30**	0.10	0.41***	0.21*	0.24**						0.80	
SATAQpeer	0.37***	0.36***	0.34***	0.19*	0.41***	0.34***	0.35***	0.47***					0.78	
SATAQmedia	0.49***	0.52***	0.44***	0.26**	0.52***	0.29**	0.31***	0.31**	0.39***				0.44	0.44
SATAQathl	0.071	0.13	0.066	0.091	0.13	0.015	0.083	0.028	0.23**	0.25**				0.87
SATAQthin	0.46***	0.48***	0.44***	0.34***	0.50***	0.19*	0.26**	0.22*	0.29**	0.41***	0.38***			0.69

PC1 loaded primarily onto the EDEQ sub-scores, BSQ and BDI, and is referred to henceforth as PSYCH. Increasing PSYCH scores represent increasing psychological concern about body shape, weight, and eating, together with an increasing tendency towards depressive symptoms. PC2 loaded primarily onto SATAQ family and peer pressures, as well as SATAQ media and RSE scores, henceforth referred to as FAMPEER. Increasing scores on FAMPEER represent an increasing tendency to internalize family, peer, and media pressure to be thin, together with reduced self-esteem. Finally, PC3 loaded primarily onto SATAQ internalization of thinness/low body fat and muscular/athletic appearance, henceforth referred to as ATHIN. Increasing scores on ATHIN represent increasing internalization of pressures to appear thin and muscular. SATAQ media pressure scores cross-loaded onto PC2 and PC3.

Next, we used PROC MIXED in SAS (v9.4) to run a linear mixed effects model to predict self-estimated BMI from group, age, actual BMI, PSYCH, FAMPEER, ATHIN, and study location. We used the Satterthwaite method to estimate degree of freedom. The final model explained 71.0% of the variance in self-estimated BMI and showed statistically significant effects of age (F1, 126 = 12.03, *p* < .001, *β* = 0.14, *SE* = 0.041), BMI (F1, 126 = 153.30, *p* < 0.001, *β* = 0.91, *SE* = 0.010), group (F1, 126 = 5.58, *p* = 0.02, *β* = 6.81, *SE* = 2.88), PSYCH (F1, 126 = 20.87, *p* < .001, *β* = 1.25, *SE* = 0.27), and FAMPEER (F1, 126 = 9.39, *p* = .003, *β* = 0.82, *SE* = 0.27). We also found a statistically significant interaction between actual BMI and group (F1, 126 = 6.66, *p* = .01, *β* = -0.31, *SE* = 0.12).

**Fig 2:**
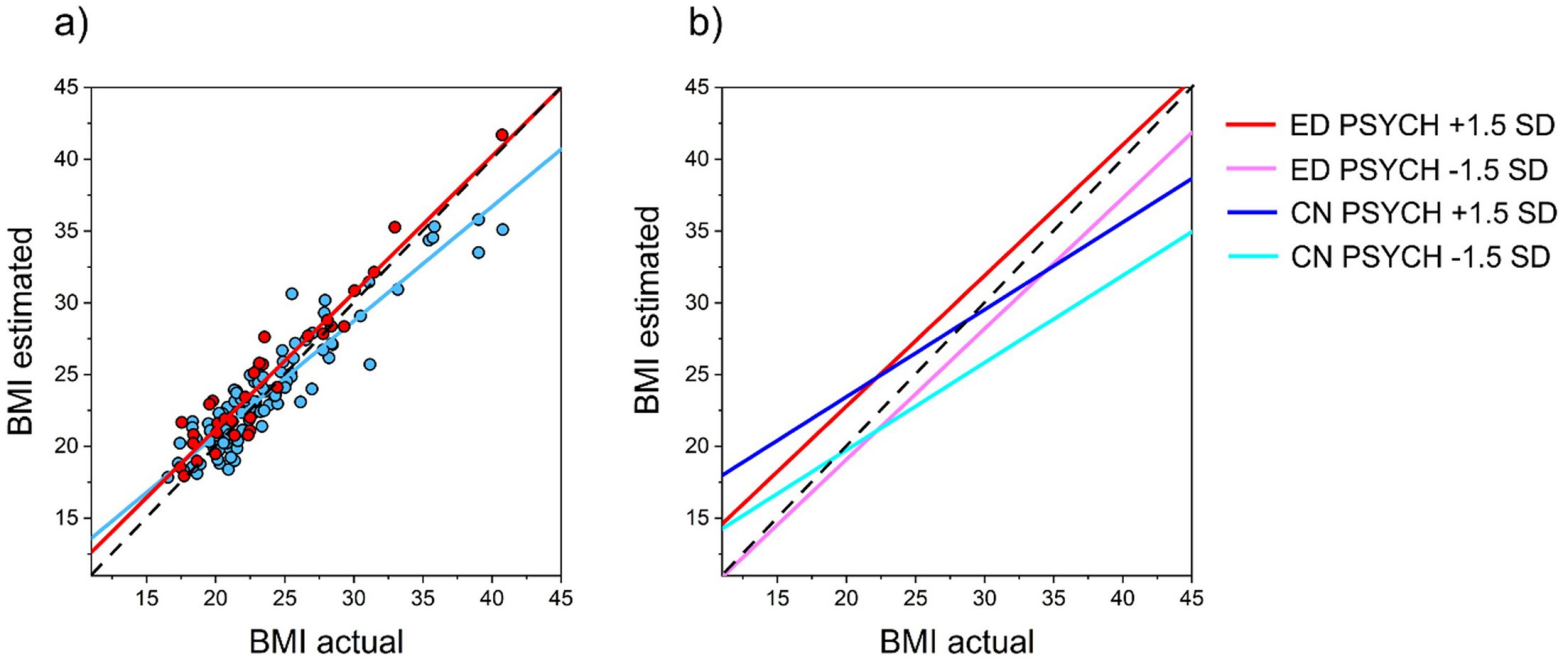
(a) The relationship between participants’ BMI (x-axis) and their subjective estimate of body size (y-axis) for women with a history of Anorexia, Bulimia, and OSFED (red) and healthy controls (blue). The effects of age, PSYCH, and FAMPEER are statistically controlled. (b) The relationship between participants’ BMI (x-axis) and fitted values of estimated body size computed from the linear mixed effects model at ±1.5 SD for the value of PSYCH for each group. In both (a) and (b), the black dashed line represents the veridical relationship between estimated and actual BMI, with a slope of 1, indicating fully accurate estimation of own body size.

There were no other statistically significant interaction terms in the model, and specifically no effect of study location. The fixed effects of ATHIN and study location were also non-significant. Critically, the regression weight for actual BMI was significantly less than 1 for the healthy controls (F1, 97 = 15.24, *p* < 0.001, *β* = 0.74, *SE* = 0.067), indicating contraction bias but not for the women with a history of eating disorders (F1, 30 = 0.15, *p* = 0.7, *β* = 0.95, *SE* = 0.12). Fig 2 illustrates the model outcome. For healthy controls (blue), Fig 2a clearly shows the positive relationship between BMI and self-estimated BMI with a slope less than 1, consistent with contraction bias. By comparison, women with an eating disorder history (red) show a regression slope close to 1 with no contraction bias. Fig 2b illustrates the independent contribution from PSYCH, whereby individuals with greater psychological concerns about their body shape/size (i.e. higher values of PSYCH) report higher self-estimated BMI in the task. Importantly, this statistically independent effect applies equally to both healthy controls and women with a history of eating disorders.

### Multivariate statistics: Separating individuals with AN and OSFED from those with BN

The key evidence for the presence of contraction bias is that the regression slope of estimated BMI on actual BMI is less than 1. To estimate this parameter reliably, the predictor variable (actual BMI) needs to have a reasonable range. As Table 2 shows, the actual BMI ranges for healthy controls and women with an eating disorder history were well matched. The initial multivariate analysis shows that the regression slope for healthy controls is indeed significantly less than 1, while the slope for women with an eating disorder history is ~1, with a statistically significant interaction between actual BMI and group.

**Fig 3:**
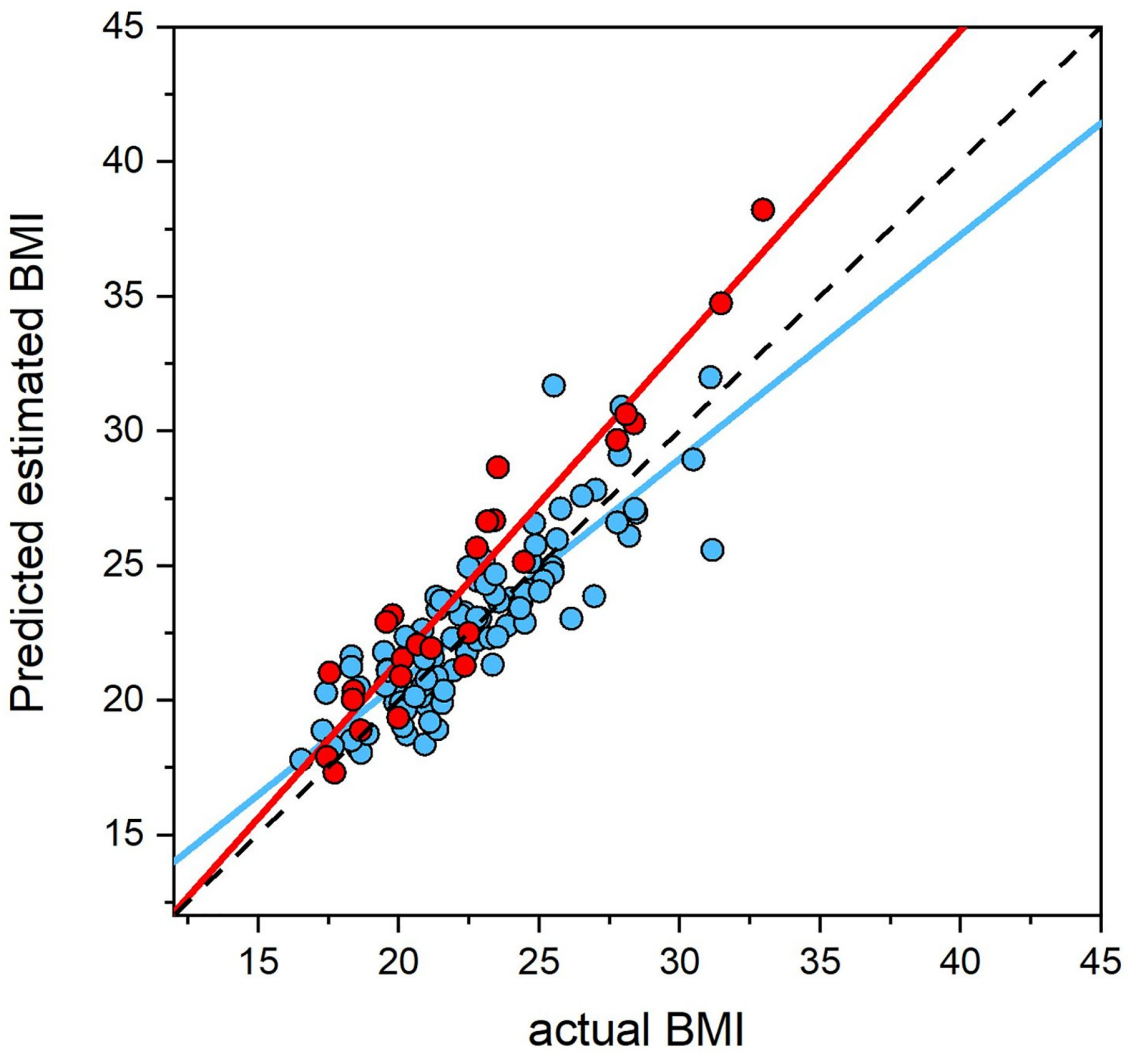
A scatterplot of the relationship between participants’ BMI (x-axis) and their subjective estimate of body size (y-axis) for women with a history of Anorexia and OSFED (red) and healthy controls (blue). The effects of age, PSYCH, and FAMPEER are statistically controlled. The black dashed line represents the veridical relationship between estimated and actual BMI, with a slope of 1.

However, we need to be sure that this result is not confounded by Simpson’s paradox [49,50]. This is the phenomenon in statistics in which a trend can appear in a large sample that disappears or reverses when sub-groups within the sample are considered separately. What subgroups might be meaningful here? The obvious possibility is that the women in this study have a history of one of three eating disorders. Moreover, differences in the way individuals with BN versus those with AN or atypical AN (i.e., OSFED here) process perceptual body image have been reported (see e.g. [34,51]). More specifically, given that the women with OSFED exhibited the atypical anorexia pattern in the current study, any differences in performance on the MoA task between women with BN versus (atypical)AN will most likely surround the third DSM 5 criterion: i.e., “Disturbance in the way in which one’s body weight or shape is experienced”, and this criterion plays a stronger role for AN than BN. Accordingly, further investigation of the current dataset revealed that the range (i.e., maximum minus minimum) for actual BMI in women with anorexia, bulimia, controls, and OSFED was: 15.52, 7.90, 24.24, and 10.55, respectively. This suggests that the BMI range for women with bulimia was potentially inadequate to make a reliable contribution to the regression coefficient for participants with an eating disorder history, and that the BMI range for controls was inappropriately large once the subgroups of eating disorder participants are considered. Therefore, we ran a second multiple regression model, excluding women with bulimia and restricted the upper BMI range for controls to ~31, reducing the actual BMI range for controls down to 14.63. Under these conditions, each of the remaining three groups (AN, OSFED, and controls) were better matched for actual BMI range. The updated model explained 68.0% of the variance in self-estimated BMI and showed statistically significant effects of: age (F1, 112 = 17.74, *p* < .001, *β* = 0.17, *SE* = 0.040), BMI (F1, 112 = 128.05, *p* < .001, *β* = 1.15, *SE* = 0.12), group (F1, 112 = 10.68, *p* = 0.001, *β* = 11.58, *SE* = 3.54), PSYCH (F1, 112 = 22.81, *p* < .001, *β* = 1.27, *SE* = 0.27), and FAMPEER (F1, 112 = 10.81, *p* = .001, *β* = 0.82, *SE* = 0.25). Critically, we still found a statistically significant interaction between actual BMI x group (F1, 112 = 12.67, *p* < .001, *β* = -0.55, *SE* = 0.15). Fig 3 shows a plot of self-estimated BMI as a function of actual BMI, predicted from this model, which excludes women with a history of bulimia. It is now clear, as was the case in Cornelissen et al. [17,18], that the slope of the regression of self-estimated BMI on actual BMI is greater than 1 (*β* = 1.18, *SE* = 0.15), indicating an accelerating pattern of over-estimation with increasing actual BMI.

As a final check in this post-hoc analysis, we wanted to know if there might be something specific about the sample of women with bulimia which could have influenced the post-hoc analysis, rather than simply removing their BMIs from the dataset of women with an eating disorder history. To address this, we randomly selected six participants from the whole eating disorder sample whose BMI fell within the same range as the bulimics and removed them. This was repeated 10,000 times, and the regression slope for BMI estimated on each occasion. This simulation produced an average regression parameter for BMI: *M* = 1.030, *SE* = 0.00084. We then ran a t-test of location to investigate whether this value was significantly different from the value estimated by removing the participants with a history of bulimia only, i.e., *β* = 1.18. The test was statistically significant (*t* = -169.78, *p* < .001), suggesting that there may be something specific about individuals with a history of bulimia leading to a reduction in the slope for the full sample of women with an eating disorder history, as shown in the first multivariate analysis.

## Discussion

This study expanded the exploration of the perceptual and attitudinal dimensions of body image, originally reported by Cornelissen et al. [17,18], by comparing individuals with a history of eating disorders to healthy controls. Our findings indicate that the perceptual aspects of body image assessment differ between these two groups. The key finding supporting this claim is the significant interaction between actual BMI and participant group in the models predicting participants’ estimates of their own BMI using the MoA task.

### Healthy controls

**Fig 4:**
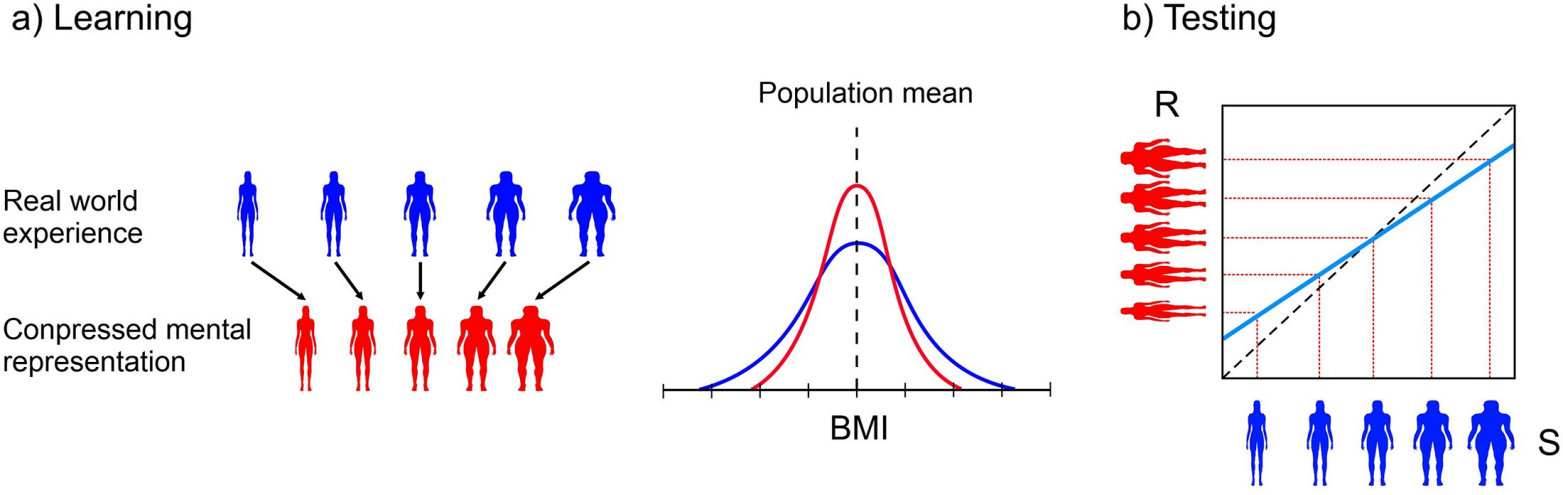
a) Cartoons illustrate bodies in a range of BMIs (blue) that people are exposed to in the real world and the mapping of these bodies onto a mental representation with a compressed range (red). This gives rise to the wider (blue) and narrower (red) distributions centred on the population average. b) Shows how the stimulus (S) response (R) relationship has a slope < 1 when participants estimate their body size. The dashed black line in the graph in b) represents veridical performance (i.e. perfect accuracy), where the slope = 1.

Consistent with prior research [19–25], healthy controls demonstrated a contraction bias in body size estimation, aligning their perception with an internal ’average’ or ’normative’ size reference distribution. This normal pattern of behaviour was consistent in both the online and laboratory-based data, as illustrated in Fig 2. According to Poulton [15], throughout our lives, we are exposed to real-world distributions of object properties. Fig 4a is an example of such a distribution for BMI (blue). The contraction bias argument posits that the mental representation of this distribution becomes compressed around the population mean, as shown by the red distribution in Fig 4a. As a result, a plot of responses (y-axis) to a range of stimuli (x-axis) in Fig 4b results in a regression slope less than one, due to this stimulus-response compression.

### Participants with a history of ED

In contrast, the clinical group, comprising individuals with current or past eating disorders, demonstrated a notably different pattern. When analysing the entire clinical group, their self-estimated BMI closely matched their actual BMI, evidenced by a regression slope close to one (see Fig 2), suggesting a relatively accurate perception of own body size across the actual BMI range. However, excluding the participants with a history of bulimia nervosa and repeating the analysis, yielded results more consistent with Cornelissen et al. [17,18], as illustrated in Fig 3. Specifically, the regression slope of estimated BMI on actual BMI had a slope > 1, with accurate estimation at low actual BMI, and systematically increasing overestimation as actual BMI increased. How should we reconcile these differences in the results? We argue that our most important finding is that both multivariate analyses revealed a statistically significant interaction between participant group and actual BMI, irrespective of the inclusion or exclusion of participants with a history of bulimia nervosa. This suggests that whatever is going on, women with a history of eating disorders perform differently from healthy controls in their perceptual judgement of body size (i.e., they do not demonstrate a normal contraction bias), indicating that disturbances in perceptual body image associated with eating disorder may persist post-recovery.

Thereafter, the key is to determine whether, in reality, the regression slope for individuals with a history of ED is equal to 1 or greater than 1. It is also important to consider the possibility that different eating disorders may be associated with different slopes. The post-hoc analysis we carried out to exclude Simpson’s paradox demonstrated that this is plausible with respect to the distinction between women with bulimia nervosa versus anorexia nervosa or atypical anorexia nervosa (i.e., OSFED, in the current study). Unfortunately, the sample size in the current study is too small to provide a definitive answer. Further research with substantially larger samples of women with eating disorder histories is therefore needed to obtain robust estimates of these regression slopes for different clinical subgroups. Nevertheless, at this stage we can offer putative explanations for the two potential outcomes to be revealed by future investigation: a) where β = 1, and/or b) where β > 1.

### Regression slope β = 1

**Fig 5:**
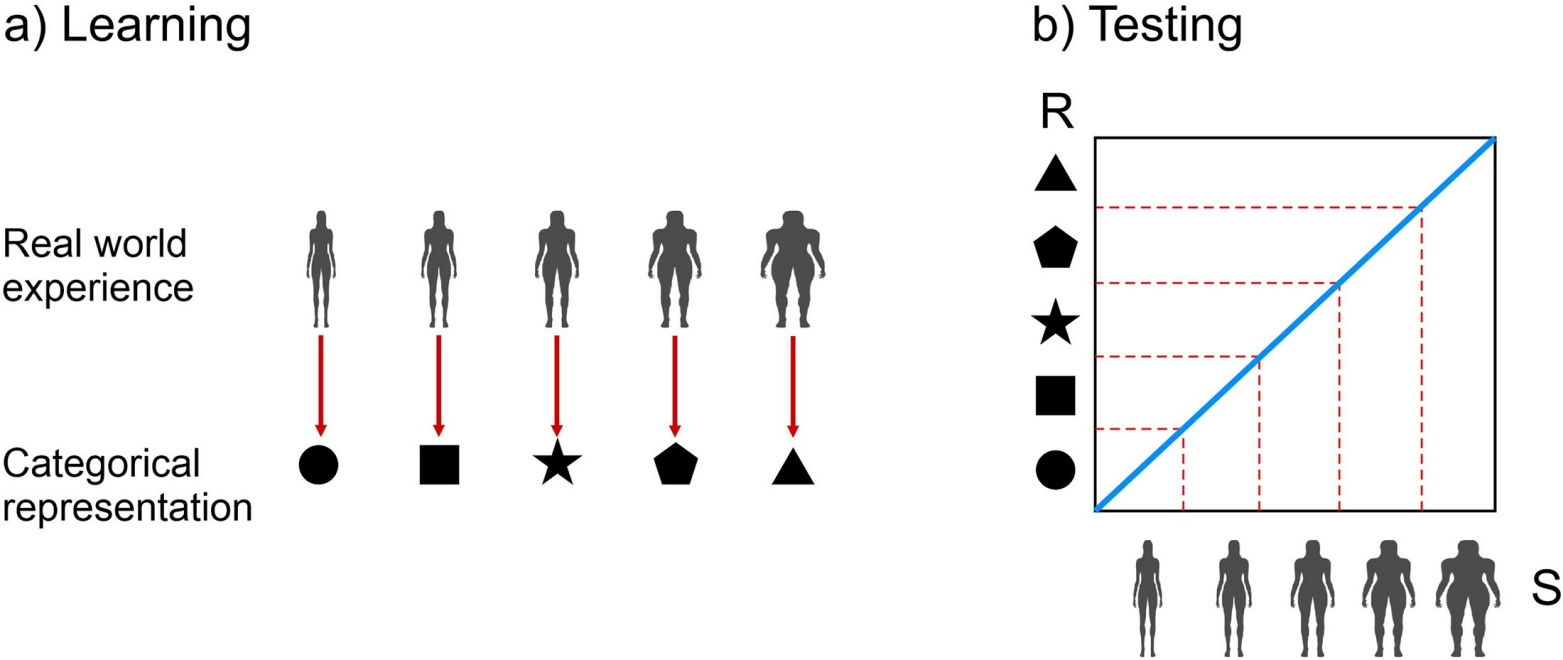
a) Cartoon illustrates how individuals with eating disorders might learn to associate narrow BMI ranges into rank orderable categories. b) Shows increasing BMIs across the stimulus range (S) are correctly mapped onto the appropriate response (R) categories, leading to accurate performance, where the slope of the regression between the two is 1.

Normal performance in human body size/shape estimation, as with any other magnitude estimation, typically exhibits contraction bias, leading to a regression slope of < 1 for the estimated BMI on actual BMI. Therefore, how could a regression slope of 1, indicating accurate self-estimation of BMI, be achieved through an ‘abnormal’ or ‘atypical’ process? One possibility is that individuals with eating disorders may develop a strategy for representing body sizes in terms of categories. Categorical perception (CP) occurs when an individual perceives a continuously varying stimulus as distinct categories rather than a smooth progression [52]. Categorical perception has been demonstrated psychophysically for face identity [53], human emotion [54], and body shape [55]. Moreover, machine learning has been applied to classify single front-view images of people into five distinct body shape categories: rectangles, triangles, inverted triangles, spoons, and hourglasses [56]. Therefore, we suggest that visible physical cues on the body may be sufficient for women with eating disorders to learn perceptual classifications of body sizes, which reliably segregate the BMI continuum into rank ordered categories, as illustrated in Fig 5. This categorical perception could enable individuals with eating disorders to discriminate between different body sizes more distinctively, and thus achieve a regression slope of 1 indicating accurate self-estimation.

### Regression slope β > 1

A slope > 1 for the regression of estimated BMI on actual BMI is consistent with our previous studies [17,18]. One possible explanation for this pattern in women with eating disorders may be the development of an expertise effect in the judgement and discrimination of low BMI bodies. Women with eating disorders often spend a great deal of time looking at low BMI bodies, including their own, but also online as part of a preoccupation with the thin ideal typically associated with the disorders [57,58]. Repeated evaluation and discrimination of low BMI bodies could lead to the development of an expertise in discerning low BMI bodies. Previous studies suggest that extensive practice in discriminating feature changes can significantly improve the ability to make fine judgements; the expertise effect [59,60]. We suggest that such an expertise effect would be specific to the low BMI bodies, as body shape changes non-linearly with increasing BMI [61]. Thus, the pattern of shape change with weight increase is different in low BMI bodies compared to larger BMI bodies, and the expertise developed by women with eating disorders would be specific to low BMI bodies and would not generalise to judging higher BMI bodies.

Evidence supporting this idea comes from Cornelissen et al. [17,18], where participants’ body size estimates were made using a yes/no task which allowed full reconstruction of the psychometric function. This allowed participants’ uncertainty (indexed by the difference limen, DL) about their body size estimates to be measured accurately. Healthy controls show a systematic increase in uncertainty which follows Weber’s law [62]. Weber’s law states that the uncertainty in judging the difference between two stimuli will be a constant proportion of their magnitude, leading to a constant Weber fraction over the stimulus range (i.e. ΔI/I = K, where I = stimulus magnitude and K = constant). This means that as a body gets heavier, it gets progressively harder to detect differences in body mass–i.e. uncertainty increases.

In contrast, Cornelissen et al. [17,18] found that women with eating disorders showed a finer discrimination of low BMI values than controls (i.e. lower DL, lower uncertainty), but this discrimination rapidly became worse than controls as the BMI of the bodies judged increased (i.e. higher DL, higher uncertainty)–their behaviour was not consistent with Weber’s law. This pattern of results suggests an expertise effect at low BMI values, but that this expertise does not generalise to higher BMI values, explaining the rapidly increasing inaccuracy of the judgements as BMI rises. However, this explanation is incomplete. It does not explain why women with eating disorders overestimate body size as BMI increases, rather than underestimating it, or just show a greater variability in estimates while maintaining the same average accuracy of estimation as the controls (i.e., a greater random variation around the mean). Instead, women with eating disorders show a rapid increase in the degree of overestimation as their BMI increases. To explain this directional shift in over-estimation as a function of increasing uncertainty, we suggest that an additional factor is at play.

**Fig 6:**
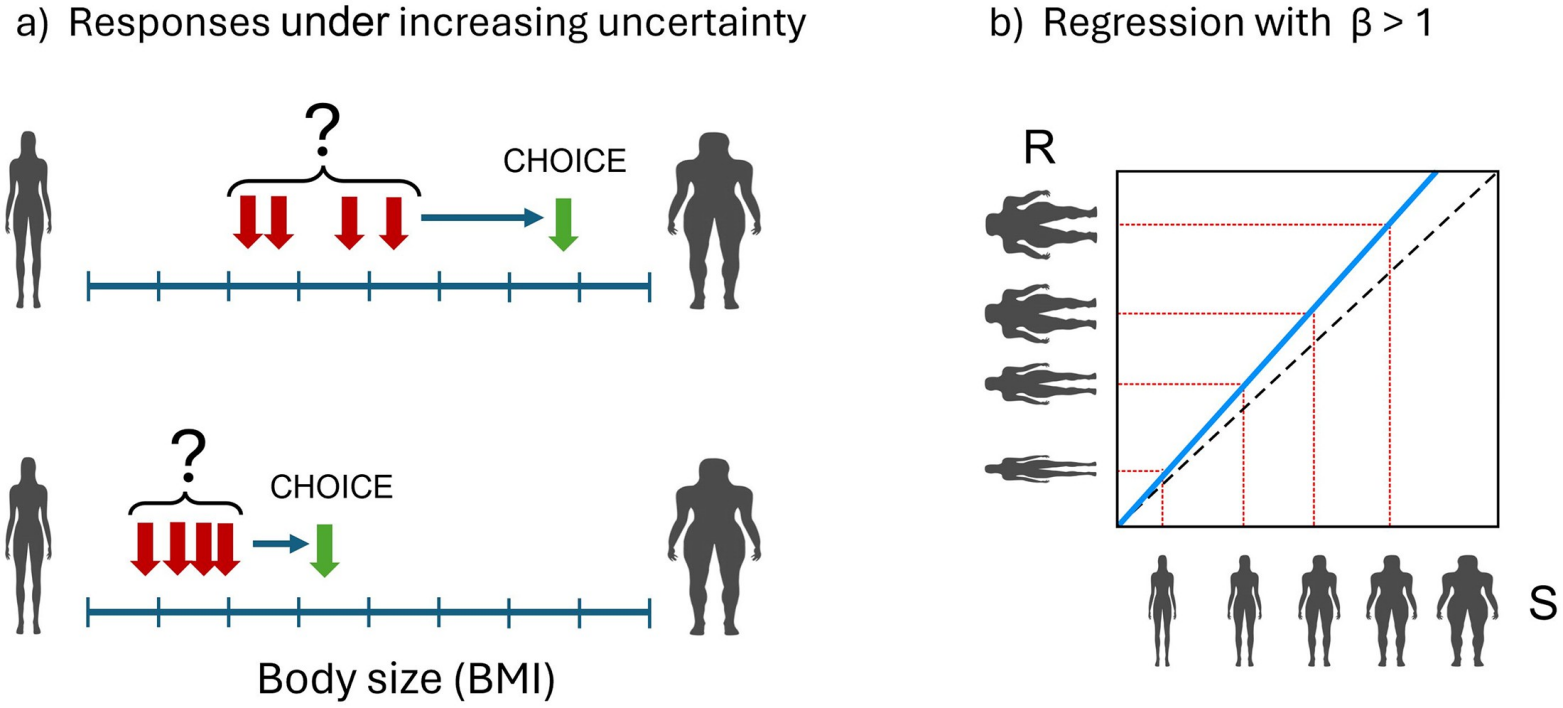
a) The lower half shows uncertainty around possible response choices (red arrows) to a lower BMI stimulus, with a small shift to the right to ensure that the final choice (green arrow) is unlikely to be incorrect. The upper half shows wider uncertainty in the possible response choices (red arrows) to a higher BMI stimulus, with a larger shift to the right to ensure that the final choice (green arrow) is unlikely to be incorrect. b) Accurate performance at lowest BMIs for stimuli in the ‘expert range’, with increasing overestimation as the right shift (green arrows) in a) systematically increases as a function of stimulus BMI, leading to a slope > 1.

Specifically, we propose that their increasing uncertainty due to the expertise effect, coupled with the documented aversion of individuals with AN to making errors in judgements [63,64] plays a key role. If one assumes that as the BMI of women with eating disorders increases and their ability to make fine discriminations between body sizes reduces due to increasing uncertainty, the range of possible estimates they could make around the actual body size increases. In the face of this increased uncertainty, their psychological concerns about their body size and an unwillingness to make mistakes may push them to choose higher BMI values–thereby avoiding errors. This putative process is illustrated in Fig 6.

### The influence of psychological factors

We indexed our participants’ psychological concerns about body image using a set of psychometric questionnaires. As in previous studies, we used a principal components analysis to extract the different psychological features from these measures [17,18]. The PSYCH component (PC1) indexed participants’ concerns about their body size, shape, weight and eating, along with depressive symptomatology. Higher PSYCH scores were associates with higher self-estimated BMI in both healthy controls and women with eating disorders.

It is important to note that the relationship between estimated BMI and actual BMI, as a function of changing PSYCH, is statistically independent and does not differ between the participant groups. Instead, PSYCH modulated the estimated BMI for any given body size, irrespective of group (see Fig 2b), and this replicates the findings of Cornelissen et al. [17,18]. The FAMPEER component (PC2) embodied increasing internalization of family, peer, and media pressures to be thin, together with lowering self-esteem. This component was also a significant and independent predictor of the degree to which participants overestimated their body size. Again, this effect was equally true for both healthy controls and women with an eating disorder history. This is consistent with previous studies that have found that internalization of family, peer, and media pressures to be thin significantly impacts feelings of body dissatisfaction and body size judgements [65,66].

Surprisingly, the ATHIN component (PC3), which embodies the extent to which a participant internalises thin and athletic body concepts, was not a significant predictor of body size estimates in the current study. Previous research has shown that internalisation of these concepts predicts judgements of attractiveness and body ideals when measured in body composition space, i.e., a higher level of internalisation leads to a preference for body ideals with less adipose and more skeletal muscle mass [67]. Therefore, future studies attempting to replicate the current study design may need to vary the body composition of stimuli, as opposed to BMI only, in order to demonstrate an influence of internalization of thin and athletic body ideals on self-estimates of body size and shape.

Interestingly, in the current data set the correlations between self-estimated body size and SATAQ thin and SATAQ athletic–which primarily drove the ATHIN factor—were r = 0.01, p = .9 and r = -0.24, p = .006, respectively. However, if these correlations were repeated, partialling out the influence of age and actual BMI, the same correlations become r = 0.38, p < .0001 and r = -0.05, p = .6, respectively. Therefore, when analysed in isolation, these two elements of the SATAQ (i.e., internalization of the thin and muscular ideals), behaved more or less as one might expect. But Table 3 also shows that SATAQ thin, in particular, was strongly correlated with the four EDEQ subscales and BSQ which contributed primarily to the PSYCH factor. Therefore, especially when actual BMI and age are controlled for (as is the case in our final linear mixed effects model), it seems plausible that the variance in self-estimated body size that would otherwise be accounted for by SATAQ thin, is instead accounted for by PSYCH.

Finally, related to psychological factors is the potential impact of linguistic effects in body size estimation. Recent research by Behrens et al. [68] asked participants to rate adjectives for their match to computer generated bodies varying in body mass index. Broadly, positive adjectives were associated with lean bodies, whereas negative adjectives were associated with obese bodies. The authors then tested how body mass index of bodies was associated with positive or negative valence of the adjectives in women with AN, atypical AN, and healthy controls and found effects indicative of weight stigmatization, but no significant differences between the groups. With respect to body size estimates of self, Behrens et al [68] also found that all participants over-estimated body-size, but that the over-estimation was significantly larger for women with typical and atypical AN than controls. However, it is important to note that the range of body sizes in all three groups, indexed by BMI, was relatively narrow. Therefore, these authors would not have been in a position to examine differences in the slopes of the relationship between self-estimated BMI versus actual BMI–the central theme of the current paper.

### In-person versus online testing

Approximately half of the data in this study was collected from participants who attended the laboratory in person, while the other half was collected online. It has been suggested that data collected online may be of lower quality than that collected in person [69,70], because online participants cannot easily get assistance if any details of the study are unclear and may not give the task their full attention [71,72]. However, although some studies have suggested small differences between the results from online and in-person studies [73,74], we found no difference in the data in our current study, nor in a previous study [21].

Thus, this study provides additional support for the utility of online testing, which allows quick and effective data collection and facilitates the recruitment of participants who might be reluctant to participate in person, such as those with mental health issues such as eating disorders. More generally, online testing may help expand participant recruitment, which frequently relies on undergraduate volunteers, potentially limiting generalisability [75–80]. Instead, recruitment through online platforms like Prolific and MTurk allows the use of stratified, randomised recruitment strategies through participant recruitment filters [21]. It also simplifies recruitment from diverse cultures and countries, expanding testing beyond Western, Educated, Industrialized, Rich, and Democratic (WEIRD) participants, as previous studies suggest there may be significant cross-cultural differences [81,82].

### Limitations

While this study provides valuable insights into the perceptual and attitudinal dimensions of body image in individuals with a history of eating disorders, the current study has some notable limitations. Despite being supported by our a priori power calculations, the sample size, particularly within the clinical group, was relatively small. While we replicated the key finding of a significant interaction between actual BMI and group, this nevertheless limited our ability to draw definitive conclusions about any differences in regression slopes between the clinical sub-groups. Future research should aim for larger and more evenly distributed sample sizes and BMI ranges across clinical subgroups to provide more robust estimates of regression weights and improve the generalizability of the findings. The study also focused primarily on BMI as a measure of body size. While BMI is a widely used metric, it does not capture other important aspects of body composition, such as fat distribution and muscle mass [83,84]. These two components play an independent role in body judgements and ideals [85,86]. Future research should incorporate more comprehensive measures of body composition to provide a fuller understanding of body image perceptions. Addressing these limitations in future research could provide a more nuanced and comprehensive understanding of the complex dynamics of body image in individuals with eating disorders, ultimately informing more effective interventions and support strategies.

Future research could also aim for subgroup analyses that account for the type of eating disorder, current status (e.g., active, recovering, recovered), and history of relapse should be conducted. Such stratified analyses would provide a more nuanced understanding of how different eating disorders, and the journey through recovery, impact perceptual and attitudinal body image. Exploring the interaction between diagnosis, recovery status, and body image could offer valuable insights into the persistence of body image disturbances post-recovery and inform targeted interventions. In a similar vein, future research should take account of effects related to sexual orientation. For example, recent research on cognitive weight bias [87] has explored the role of body weight and shape concerns in gay men and bisexual women. The authors demonstrated the vulnerability of gay men and bisexual women towards such cognitive biases about their own bodies.

## Conclusion

The current study adds valuable insights into the complex dynamics of body image in women with a history of eating disorders. The observed differences between the clinical (including or excluding BN participants) and control groups underscore the significant impact of eating disorders on perceptual body image, even if the exact clinical pattern remains to be fully elucidated.
